# Action dynamics in multitasking: the impact of additional task factors on the execution of the prioritized motor movement

**DOI:** 10.3389/fpsyg.2015.00934

**Published:** 2015-07-06

**Authors:** Stefan Scherbaum, Caroline Gottschalk, Maja Dshemuchadse, Rico Fischer

**Affiliations:** Department of Psychology, Technische Universität DresdenDresden, Germany

**Keywords:** action dynamics, mouse movements, crosstalk, dual task, task shielding, cognitive control, conflict adaptation

## Abstract

In multitasking, the execution of a prioritized task is in danger of crosstalk by the secondary task. Task shielding allows minimizing this crosstalk. However, the locus and temporal dynamics of crosstalk effects and further sources of influence on the execution of the prioritized task are to-date only vaguely understood. Here we combined a dual-task paradigm with an action dynamics approach and studied how and according to which temporal characteristics crosstalk, previously experienced interference and previously executed responses influenced participants' mouse movements in the prioritized task's execution. Investigating continuous mouse movements of the prioritized task, our results indicate a continuous crosstalk from secondary task processing until the endpoint of the movement was reached, although the secondary task could only be executed after finishing execution of the prioritized task. The motor movement in the prioritized task was further modulated by previously experienced interference between the prioritized and the secondary task. Furthermore, response biases from previous responses of the prioritized and the secondary task in movements indicate different sources of such biases. The bias by previous responses to the prioritized task follows a sustained temporal pattern typical for a contextual reactivation, while the bias by previous responses to the secondary task follows a decaying temporal pattern indicating residual activation of previously activated spatial codes.

## Introduction

Multitasking seems to be ubiquitous in today's world. The execution of multiple tasks at the same time, however, runs the risk of the prioritized task's performance being affected by the additional tasks. For example, even highly practiced and prioritized driving performance might suffer from additional task execution (e.g., Levy et al., 2006; Strayer and Drews, 2007).

In the present study it was thus asked to which extent continuous motor movements of a prioritized task are affected by determinants of a multitasking context (e.g., programming of a subsequently executed motor task). To study how a prioritized task (e.g., Task 1: a number magnitude judgment) is influenced by simultaneous processing of additional task components (e.g., Task 2: a tone frequency judgment) most experiments use dual-task paradigms in which (1) the stimulus (S2) of Task 2 is presented in various intervals (stimulus onset asynchronies, SOA) after the stimulus (S1) of Task 1 (Pashler, 1994) and (2) both tasks share dimensional overlap (Navon and Miller, 2002). In these settings, many studies reported that programming of the response (R1) in Task 1 is affected by simultaneous programming of the response (R2) in Task 2, reflected in so-called crosstalk effects. For example, Hommel (1998a) demonstrated crosstalk effects on RT in Task 1 (RT1) when the response codes of Task 1 and 2 overlapped, i.e., responding to colors in Task 1 with a left or right key-press and to letters in Task 2 by saying “left” and “right.” In this case, RT1 decreased when Task 2 indicated the same response category, while it increased when Task 2 indicated a different response category.

Although the amount of between-task interference, reflected in crosstalk effects, has often been taken to indicate the effectiveness of cognitive control in shielding the prioritized task processing i.e., small crosstalk effects reflecting strong task shielding (Logan and Gordon, 2001; Fischer and Hommel, 2012; Plessow et al., 2012; Fischer et al., 2014), the locus and temporal dynamics of crosstalk effects are to date only vaguely understood. In addition, while most studies investigated how response programming in one task affects response programming in another, only few studies targeted execution-related interference between tasks. For example, Bratzke et al. (2009) used continuous motor movements in Task 1 and found propagation effects of movement distance in Task 1 on choice RT in Task 2, indicating Task 1 motor execution-related interference in Task 2. In a similar setup, Ulrich et al. (2006) found that prolonging response-execution in Task 1 also increased choice RT on Task 2. While these studies investigated effects of continuous motor movements on additional task processing, we pursued the opposite approach by focusing on the quality of the prioritized continuous motor execution and crosstalk effects due to additional task processing on these movements. More specifically, we applied a crosstalk approach to test in which time windows and by which factors the continuous motor execution of a prioritized Task 1 is affected. For this we designed a dynamic dual-task paradigm, in which participants had to move a computer mouse to respond in both tasks. Importantly, a continuous analysis of mouse movements allows first, to track accuracy/quality of the movement parameter and second to determine the temporal characteristics of the influencing factors (Spivey et al., 2005; Freeman et al., 2008; Scherbaum et al., 2010).

**Figure 1:**
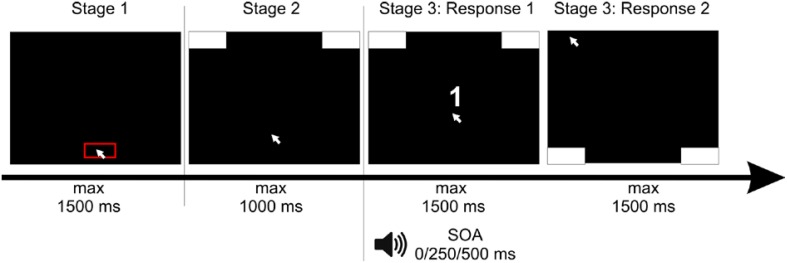
**Setup of the experiment: Participants had to click with the mouse cursor into a red box at the bottom of the screen**. After clicking, response boxes appeared at the upper edge of the screen and participants had to move the cursor upwards, in order to start the trial. After reaching a movement threshold, the stimulus of the first task—a white number—appeared. The second stimulus, a tone, was presented with a stimulus onset asynchrony (SOA) of 0/250/500 ms. For the response to the first stimulus, participants had to move the mouse cursor to the left or the right upper response box as indicated by the number. Afterwards—for the response to the second stimulus—participants had to move the mouse cursor to the left or the right bottom response box as indicated by the tone.

We hypothesized at least three important factors to determine motor execution in Task 1. First, we predict that *simultaneous programming of an additional motor response* affects the execution of the prioritized motor movement: interference and hence deviations of the prioritized movement could be expected if the additional motor response points into a different direction as the prioritized movement. Participants responded to the magnitude of a presented number (S1) by moving the mouse to a pre-defined target region (see Figure 1). To ensure R2 programming while executing Task 1, S2 (high vs. low tone) was presented shortly with different SOAs following S1. Both tasks were performed sequentially using the same response device. R1 was given by moving the mouse toward target regions at the *upper* left and the *upper* right of the screen, while R2 was given by subsequently moving the mouse toward target regions at the *lower* left and the *lower* right of the screen. The brief presentation of S2 required encoding and possibly programming of R2. Yet, the execution of R2 was not to start until execution of R1 was completed. If R2 programming starts prior to completion of R1 execution, programming R2 that entails opposite directional movement parameters (e.g., a spatial code for the target region on the right side of the screen) should critically affect the quality of the continuous motor execution of R1 (e.g., movement in direction of the left target region). Importantly, and in contrast to previous crosstalk studies employing discrete button presses, the continuous performance measure allows determining whether and to which time point R2 programming affects the movement of R1. This is not trivial, as findings from single tasks indicate time-sensitive profiles of interference. Whereas, in the Simon task interference effects decrease over time due to either decay or suppression of conflicting information (Hommel, 1994; Stürmer et al., 2002; Band et al., 2003; Scherbaum et al., 2010), in the Stroop task and the Flanker task, the opposite temporal pattern was observed (Pratte et al., 2010; Ulrich et al., 2015). In addition, R1 movement execution might reach a ballistic dynamic in the direction of the aimed target region at which it might become immune to influences of additional Task 2 processing.

Second, in a recent study, we found that *previously executed responses* influenced motor movement parameter in the current trial (Scherbaum et al., 2010). At the start of a trial, participants showed a movement bias in the direction of the previously executed response. In the present dual-task situation, two responses from the previous trial could influence Task 1 execution: the previous response to Task 1 and the previous response to Task 2, respectively. Because the movement pattern of R2 (downward movement) differs substantially to the movement pattern of R1 (upward movement), a bias on the current R1 by pervious R2 could be driven by residual activation of the spatial code (left vs. right target region) of the previous R2. This residual activation should drop off quickly after response execution and hence, one could expect this influence to decay quickly.

Given the similarity of previous and current R1 (both upward movements) we assume that a bias may consist of the reactivation of the entire motor program including the previously targeted spatial code. This reactivation of the previously applied motor program should be reflected in a more sustained influence on the current response to Task 1. Both influences and the described temporal patterns can be detected by the analysis of mouse movements.

As a third influential parameter on current prioritized movement execution we hypothesize that the extent of *between-task interference in the previous trial* (i.e., the level of crosstalk in trial_N−1_) will determine shielding of the currently executed motor response from crosstalk. This assumption is derived from the influential conflict monitoring theory (Botvinick et al., 2001) which proposes that an experienced response conflict triggers a recruitment of cognitive control to optimize subsequent performance. As a consequence, interference effects are usually reduced when following a conflict (Gratton et al., 1992; Stürmer et al., 2002; Egner and Hirsch, 2005; Ullsperger et al., 2005). In a dual-task context, crosstalk interference from Task 2 onto Task 1 processing can be interpreted as conflict that in turn shows sequential dependencies (e.g., Fischer et al., 2014). Hence, it is conceivable that the present form of crosstalk in a continuous motor execution task leads to similar sequential modulations of interference. Demonstrating trial-to-trial modulations of dual-task specific crosstalk effects extends the idea of conflict adaptation to yet another form of conflict. This is not trivial, as conflicts in single tasks usually contain task-irrelevant features/stimuli that can be suppressed by mechanisms of selective attention. Dual-task processing, however, differs considerably as all features/stimuli are task-relevant and a simple selective attention mechanism might not be adaptive (see also Fischer et al., 2014).

Furthermore, by applying continuous motor movements to study these modulations in dual-task situations, our study extends previous studies investigating temporal dynamics of congruence sequence effects (e.g., Notebaert et al., 2006; Mayr and Awh, 2009; Egner et al., 2010)—however, the study of continuous movements enables a within-trial approach yielding precise temporal patterns by which previous interference affects current Task 1 execution.

## Methods

### Participants

Twenty students (17 female, mean age = 23.52 years, *SD* = 4.41) of the Technische Universität Dresden took part in the experiment^1^. All participants had normal or corrected to normal vision. They received class credit or 5 € payment.

### Ethics statement

The study was approved by the institutional review board of the Technische Universität Dresden and conducted in accordance to ethical standards of the 1964 Declaration of Helsinki and of the German Psychological Society. All participants were informed about the purpose and the procedure of the study and gave written informed consent prior to the experiment. All data were analyzed anonymously.

### Apparatus and stimuli

Target stimuli in Task 1 (numbers 1–4 and 6–9) were presented in white on a black background in the center of on a 17 inch screen running at a resolution of 1280 × 1024 pixels (75 Hz refresh frequency). S1 had a width of 6.44°. Response boxes (11.55° in width) in Task 1 were presented at the top left and top right of the screen. S2 were sine tones (low: 440 Hz, high: 880 Hz, sampled at 44,100 Hz), presented for 200 ms binaurally via headphones. Response boxes (11.55° in width) in Task 2 were presented at the bottom left and bottom right of the screen.

For presentation, we used Psychophysics Toolbox 3 (Brainard, 1997; Pelli, 1997), Matlab 2006b (the Mathworks Inc.), and Windows XP. Tones were presented via the Portaudio driver on high precision ASIO enabled soundcards. Responses were carried out by moving a computer mouse (Logitech Wheel Mouse USB), sampled with a frequency of 92 Hz.

### Procedure

After onscreen instructions and demonstration by the experimenter, participants practiced 20 trials, followed by the main experiment. The experiment consisted of four blocks and 1028 trials overall (see Design).

Each trial consisted of three stages (see Figure 1). In the first stage, participants had to click at a red box (11.55° in width) at the bottom of the screen within a deadline of 1500 ms. This served to produce a comparable starting area for each trial. After clicking within this box, the second stage started and two response boxes at the right and left upper corner of the screen were presented. Participants were required to start the mouse movement upwards within a deadline of 1500 ms. We chose this procedure forcing participants to be already moving when entering the decision process to assure that they did not decided first and then only executed the final movement. Hence, only after moving at least 4 pixels in each of two consecutive time steps, the third stage started containing the actual tasks one and two. The target stimulus of Task 1—the number—was presented. The stimulus of Task 2—the tone—was presented with a stimulus onset asynchrony (SOA) of 0, 250, or 500 ms relative to S1.

To minimize “noise” in the data (due to incompatible stimulus-space representations (e.g., Dehaene et al., 1993), we used a constant spatial-compatible mapping between stimuli and responses for all participants. More precisely, for the first task, participants were instructed to respond to the number by moving the cursor into the upper left response box for digits smaller than five and to the upper right response box for digits larger than five. After giving this response to Task 1, participants executed their response to Task 2 by moving the cursor into the bottom left response box for a low tone and to the bottom right response box for the high tone. The number and the tone either indicated the same side of response (congruent condition) or opposite sides (incongruent condition).

The trial ended after moving the cursor into the respective response boxes or within a response deadline of 1500 ms in each task (see Figure 1). If participants missed deadlines of one of the three stages, the next trial started with the presentation of the red start box. Response times (RT) were measured as the time to reach the respective response box, reflecting the interval between the onset of the target stimulus (number in the first task, tone in the second task) and reaching the response box (top ones for the first task, bottom ones for the second task) with the mouse cursor.

### Design

Across trials, we varied the following independent variables: for the current trial, *number_N_* (1,2,3,4,6,7,8, and 9) and *tone_N_* (low/high), and for the previous trial, *number*_*N*−1_ and *tone*_*N*−1_. The sequence of trials was balanced within each block by pseudo randomization resulting in a balanced trial*_N_* (16) × trial _*N*−1_(16) = 256 trials transition matrix (+1 trial to conclude the sequence of balanced transitions) for each of four blocks of trials, resulting in 1028 trials overall. On this balanced sequence of trials, the *SOA* between number and tone was distributed balanced across *congruency_N_* and *congruency*_*N*−1_ by pseudo randomization. Overall, this leads to a 2 (*congruency_N_*) × 2 (*congruency*_*N*−1_) × 3 (*SOA*) design with 85–86 trials per condition.

### Data preprocessing

We excluded trials missing deadlines or containing erroneous responses and the trial following erroneous responses in one or both of the two tasks (15.47%). For all analyses of discrete measures, Greenhouse-Geisser adjustments were applied when appropriate.

For the analysis of mouse movements, we aligned all movements for a common starting position and normalized each movement to 100 equal time slices^2^ (Spivey et al., 2005; Scherbaum et al., 2010). To quantify the deviation of mouse movements from the shortest path to the target box, we subtracted X-coordinates of each movement from an ideal X-coordinate line.

## Results

### Response times in task 1 (RT1)^3^

**Figure 2:**
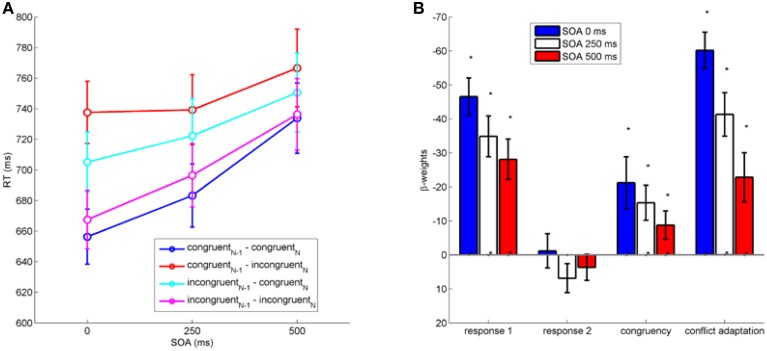
**(A):** RT1 as a function of stimulus onset asynchrony (SOA in ms) between the stimulus of Task 1 and Task 2. **(B)**: Results of multiple regression analysis on RT1 (in ms) with all hypothesized factors. Negative weights indicate a decrease in RT. Error bars represent standard errors of the mean. Asterisks indicate significant differences from zero at the 0.05 level (see main text).

We first analyzed the impact of between-task crosstalk in the current trial (*congruency_N_*) and the between-task crosstalk of the previous trial (*congruency*_*N*−1_) on RT1 (see Figure 2A). A repeated measures analysis of variance (ANOVA) on RT1 with the factors *congruency_N_, congruency_N−1_*, and *SOA* revealed significant crosstalk from Task 2 to Task 1 as expressed in the main effect of *congruency_N_* on RT1, *F*_(1, 19)_ = 11.61, *p* < 0.01, η^2^_*p*_ = 0.38. RT1 were shorter when the present trial was congruent (709 ms) than incongruent (724 ms). Post-conflict trials were slightly faster (713 ms) compared to trials following congruent trials (720 ms) as indicated by the significant factor *congruency*_*N*−1_, *F*_(1, 19)_ = 6.32, *p* < 0.05, η^2^_*p*_ = 0.25. A main effect of *SOA* indicated larger RT1 (692, 710, 747 ms) with increasing SOA, *F*_(2, 38)_ = 33.52, *p* < 0.001, η^2^_*p*_ = 0.64.

Furthermore, there was a significant two-way interaction *congruency_N_*x *congruency*_*N*−1_, *F*_(1, 19)_ = 77.53, *p* < 0.001, η^2^_*p*_ = 0.8, reflecting a conflict adaptation effect that was also present for all individual SOA levels (individual ANOVAs for each SOA, all *ps* < 0.01). Yet, the significant three-way interaction between *congruency_N_*× *congruency*_*N*−1_ × *SOA* on RT1 shows that the expression of conflict adaptation varied across SOA levels, *F*_(2, 38)_ = 11.49, *p* < 0.001, η^2^_*p*_ = 0.38 (see Figure 2A). No other interactions reached statistical significance (all *p* > 0.3).

To establish a comparison for the continuous regression analysis as performed on mouse movements (see next section), we studied the full pattern of hypothesized effects with respect to SOA by performing regression analysis on RT1. We performed regression separately for each SOA with regressors for all four hypothesized, namely *congruency_N_, conflict adaptation (congruency_N_ × congruency_N−1)_, first response in previous trial* (current R1 as repetition or switch of previous R1), and *second response in previous trial* (current R1 as repetition or switch of previous R2). All regressors were normalized to a range of [0, 1]. To exclude multicolinearity, we checked *variance inflation factors* to stay below 1. We tested the resulting 12 beta-weights (3 SOA × 4 regressors) for statistical significant influence by *t*-tests against zero.

Results (see Figure 2B) show significant beta-weights across all SOA for *congruency_N_*(all β < −9, all *t* < −2.10, all *p* < 0.05), *conflict adaptation* (all β < −23, all *t* < −3.154, all *p* < 0.05), and first *response in previous trial* (all β < −28, all *t* < −4.760, *p* < 0.05), but not for second *response in previous trial* (all *p* > 0.12). Hence, congruency within a trial, conflict adaptation, and a repetition of the previous response in Task 1 significantly influenced the response in the current trial across all SOA.

### Mouse movements in task 1

In the next step we analyzed mouse movements to investigate the temporal patterns of the different influences in dependence of the *SOA*. To this end, we performed time continuous multiple regression (Notebaert and Verguts, 2007; Scherbaum et al., 2010; but see Mirman et al., 2008 for a multilevel approach) on the deflection of mouse movements on the (horizontal) X-axis (see Supplement Figure 2): For each trial, we calculated deflection as the difference of the real movement and a straight line from the start-point to the end-point of the real movement (a hypothetical direct movement). Compared to movements on the X-axis, this measure removes random variance resulting from different start- and end-points of movements and instead focusses on the deviation of the movement away from an ideal movement due to influences during movement execution. Compared to movement-angles (Scherbaum et al., 2010), deviation is more robust to noise, as it integrates influences across time, though at the cost of temporal resolution.

For regression on this continuous measure of deflection, we used the same regressors as for RT1, namely *congruency_N_, conflict adaptation (congruency_N_ × congruency_N−1)_, first response in previous trial* (current R1 as repetition or switch of previous R1), and *second response in previous trial* (current R1 as repetition or switch of previous R2). For each time slice, we calculated a multiple regression analysis (100 time slices 
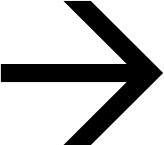
 100 multiple regressions analyses) with the four defined regressors, yielding four time-varying beta weights (4 weights across 100 time slices) for each participant. For each of these four beta-weights, we computed grand averages representing the time-varying strength of influence curve for each predictor. To detect significant temporal segments of influence, we calculated *t*-tests against zero for each time step of these beta-weights (Scherbaum et al., 2010; Dshemuchadse et al., 2012), compensating for multiple comparisons of temporally dependent data by only accepting segments of more than 10 consecutive significant *t*-tests (see Appendix for a Monte Carlo analysis on this issue, based on Dale et al., 2007).

**Figure 3:**
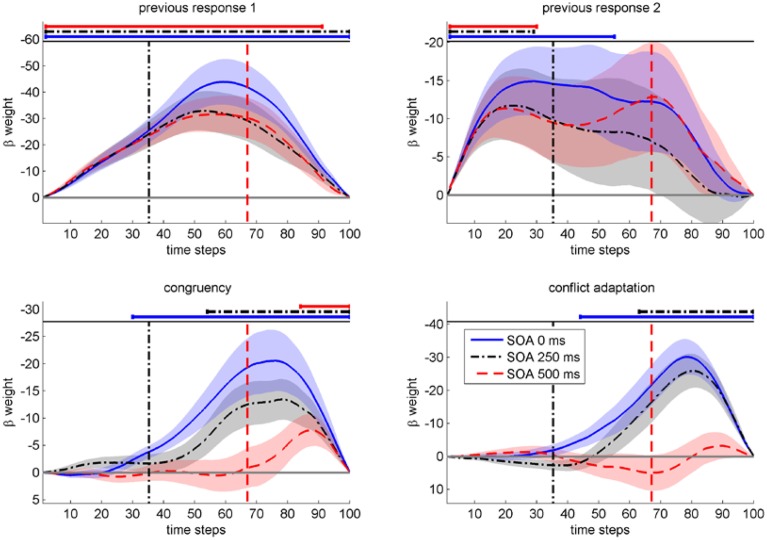
**Mean beta weights from continuous regression analysis on the deflection of mouse movements (in pixels) as a function of time steps (normalized time)**. Each regressor for each of three SOAs (in ms) is plotted in separate panels. The SOAs' time segment relative to average RT1 is indicated by the respective vertical lines. Negative beta-weights indicate a support of movement into the correct direction (smaller deflection to the incorrect target box). Shaded areas indicate standard errors.

As can be seen in Figure 3 and Table 1, *congruency_N_*showed a significant influence across all SOAs and the temporal onset of significant influence by *congruency_N_* strictly followed the SOA. Furthermore, *conflict adaptation* was only present for the first two *SOA*s, with a slight time-lag to the onset of *congruency_N_*. A long lasting influence of first *response in previous trial* was present across all SOA, while second *response in previous trial* influenced mouse movements, in contrast to RT1, but only at the start of the trial, decaying quickly in the course of the trial^4^.

**Table 1:** **Significant temporal segments (normalized time) from continuous regression analysis and respective mean RT1**.

**Regressor**		**SOA 0 ms**	**SOA 250 ms**	**SOA 500 ms**
Congruency_n_	Time steps	30–100	54–100	84–100
	M(RT)	207–691 ms	383–710 ms	627–746 ms
Conflict adapation	Time steps	44–100	63–100	–
	M(RT)	304–691 ms	447–710 ms	–
First response _N-1_	Time steps	2–100	2–100	2–91
	M(RT)	14–691 ms	14–710 ms	15–679 ms
Second response _N-1_	Time steps	2–55	2–29	2–30
	M(RT)	14–380 ms	14–206 ms	15–224 ms

These results confirm our expectation that information from the previous tasks and from the current Task 2 influenced the execution of Task 1. Furthermore, crosstalk from Task 2 on Task 1 was not limited to a specific stage of Task 1 execution, but was present for all SOA and followed the timing of the arrival of information from Task 2 as reflected in the SOA.

## Discussion

In the present study we investigated to which extent continuous motor movements of a prioritized task (Task 1) are affected by determinants of a multitasking context, namely crosstalk from a secondary task (Task 2), previously executed responses of Task 1 and Task 2, and the extent of previously experienced interference between Task 1 and Task 2 (crosstalk in trial N-1). We found evidence for an influence of all four factors, following specific temporal patterns.

First, the results from RT and mouse movements indicate that Task 1 execution is influenced by crosstalk through the information necessary to program the response of Task 2 (Hommel, 1998a; Logan and Schulkind, 2000; Fischer et al., 2007). While the analysis of RT1 indicated crosstalk to weaken with increasing SOA, the analysis of mouse movements revealed that crosstalk strictly followed the timing of the onset of the information for Task 2 as determined by the SOA. For the shortest SOA, the influence of crosstalk started the earliest and accumulated most in the deflection of mouse movements. For the longest SOA, the influence of crosstalk was limited to the final part of the movement and could accumulate only shortly. Thus, while varying in degree, crosstalk was not limited to specific critical time-windows that might be related to certain processing stages, i.e., response selection and/or movement execution of Task 1. The finding of crosstalk on Task 1 movement execution is not trivial. First of all, although the movement of the mouse itself started the trial (with S1 presentation) so that R1 programming was forced to occur online during movement, it could have been conceivable that the movement becomes a ballistic process at some point which renders it insensitive to influences of additional stimulus encoding and classification. In contrast, however, we could show that throughout the entire movement period crosstalk from additional Task 2 processing affected the movement quality in the prioritized task—even for the longest SOA, we found crosstalk, as indicated by regression analysis of RT1 and mouse movements. Furthermore, even though the execution of both tasks was temporally segregated (due to using the same response device for Task 1 and Task 2) crosstalk did occur whenever S2 was presented. This shows that the brief presentation of S2 resulted in an immediate stimulus feature encoding and response selection process that interfered with motor execution in Task 1. Put differently, despite the mouse-paradigm-inherent sequential motor execution, crosstalk from Task 2 onto Task 1 could not be prevented. Since R1 and R2 movements were executed in different vertical directions (upwards for R1, downwards for R2), one could assume that the found crosstalk does not stem directly from the programming of movements, but from the spatial (horizontal) codes for the target areas (left/right), that overlapped between Task 1 and Task 2.

Second, the analysis of RT1 and mouse movements yielded different results about the influence on R1 by previously executed responses in Task 1 and Task 2. The analysis of RT1 only revealed an influence of the previous response in Task 1, while mouse movements revealed an influence of the previous response in both Task 1 and Task 2. This indicates that RT as a discrete measure was not as sensitive as mouse movements and missed the smaller effects of the previous R2. Mouse movements further revealed distinct temporal patterns for both influences. That is, the previous response to Task 1 led to a strong and sustained influence which can be interpreted as a retrieval of the previously activated response by the context of Task 1 (cf. Hommel, 1998b; Hommel et al., 2002). Since this effect was present across the whole trial, it was also reflected in RT1. The previous response to Task 2 led to a weaker and quickly decaying influence. This could be interpreted as a passively decaying residual activation of the response executed directly before the current R1. Notably, the differences in the exact movements of R1 (upwards) and R2 (downwards) suggests that this residual activation stems from spatial codes used for programming R2, but not from the completely programmed movement of the previous R2 itself. Since the effect decayed quickly, it was not reflected in RT1, indicating the advantage of analyzing the continuous data. The finding of these two effects from previously executed responses also sheds light on a similar effect found in single task situations (Scherbaum et al., 2010, 2013): here, the strength of the found response repetition bias might result from an inseparable mixture of the retrieval and residual activation of the same response in the previous trial.

Third, congruence relations in the previous trial affected crosstalk in the current trial. More specifically, previous conflict reduced the current impact of Task 2 processing on Task 1. Therefore, experiencing crosstalk resulted in increased levels of prioritized task shielding to protect Task 1 processing from Task 2 interference that could be compared to conflict adaptation in single task situations (Botvinick et al., 2001).

Our findings support the view of a continuous process of scheduling and capacity sharing (Tombu and Jolicœur, 2003). The crosstalk between Task 1 and 2 and the influence of previous interference indicate flexible task shielding of Task 1 from Task 2 following a pattern similar to conflict adaptation in single task situation (Botvinick et al., 2001). The effects of conflict adaptation indicate that task shielding can be parameterized by previous experience of crosstalk interference (Fischer et al., 2014; compare e.g., Logan and Gordon, 2001). However, adjustments to task shielding showed a time-lag leading to conflict adaptation in mouse movements being only present for the first two SOAs. For the longest SOA, the temporal pattern shows an onset of conflict adaptation that fails to reach significance before the end of R1.

The continuous nature of our task might have supported the flexible time sharing compared to the usual key-press-based setups. We forced participants to start the movement of R1 before S1 was presented and this could have forced participants to choose a continuous processing mode that might not be chosen in a key-press based paradigm. While this procedure was necessary to ensure that response selection is reflected in the movement of R1, one could also argue that most actions in the real world demand the continuous adaptation of response movements to occurring stimuli and hence, our results imply a higher ecological validity compared to the strongly constrained key-press setups found in other studies.

Notably, our study is not the first one to apply continuous movements to respond to Task 1. However, previous studies used the continuous movements in Task 1 mainly to influence responding in Task 2 (e.g., Ulrich et al., 2006; Bratzke et al., 2008, 2009). An important variable in these studies was movement distance in Task 1, leading to higher movement times of R1. Especially for long movement distances, these studies found increasing Task 1 RT in dependence of the SOA, comparable to the results of the current study (although focusing on the consequences on Task 2; e.g., Bratzke et al., 2009).

A negative side-effect of our focus on Task 1 and the chosen response setup of our paradigm is that responses in Task 2 are hard to interpret. In the case of incongruent Task 1 and Task 2 responses, participants had a longer way to reach the opposite response box in Task 2 and hence longer RT *per se*. However, what could be taken from the pattern of RT in Task 1 and Task 2 is that our manipulation of SOA was effective. If higher SOA had shown smaller slopes in RT of Task 2 (see Supplement), it would have been possible that Task 2 information was too late to influence Task 1 (compare e.g., Ulrich et al., 2006).

Concluding, the dynamic investigation of Task 1 execution in a dual-task setting yielded three findings: First, the crosstalk from Task 2 interfered with Task 1 execution. Although this influence was clearly dependent on the temporal proximity between S1 and S2 presentation, the impact of the influence onto Task 1 processing was continuous, i.e., irrespective of any critical windows of influence. This indicates a continuous process of task execution that does not end in a ballistic automatic movement, but is prone to interference until reaching its final destination; second, the modulation of crosstalk by previous interference indicates a flexible adaptation of task-shielding; and third, the execution of Task 1 was also influenced by previously executed responses of both, Task 1 and Task 2—however, these influences showed different temporal patterns indicating a sustained reactivation of the previous response of Task 1 and a decaying residual activation that is most likely related to the spatial codes of the previous execution of Task 2.

## Author contributions

This research was supported by the German Research Foundation (DFG grant SCH1827/11 to SS and DFG grant SFB 940/1 Project A3 to RF). The funders had no role in study design, data collection and analysis, decision to publish, or preparation of the manuscript. No additional external funding was received for this study.

## Funding

We acknowledge support by the German Research Foundation and the Open Access Publication Funds of the TU Dresden.

### Conflict of interest statement

The authors declare that the research was conducted in the absence of any commercial or financial relationships that could be construed as a potential conflict of interest.

## Footnotes

^1^Descriptive data of one student participant was lost after data collection—hence, gender and age describe data of 19 participants. The experimental data are reported completely for all 20 participants.

^2^At 92 Hz sampling frequency, 100 samples correspond to an RT of 1087 ms. This means that all trials below this RT, including trials of average RT (*M* = 682 ms, *SE* = 19 ms, 62 samples at 92 Hz), are stretched to 100 samples. Only trials longer than 1087 ms (5.7% of all trials) are compressed.

^3^For RT2, please see the Supplementary Material and the Supplementary Figure 1.

^4^These analyses did not change qualitatively when removing all trials with RT lower than 500 ms.
