# Supplementary material for: Action dynamics in multitasking: the impact of additional task factors on the execution of the prioritized motor movement
Source: Front Psychol. 2015 Jul 6;6:934. doi: 10.3389/fpsyg.2015.00934 (PMC4491597; doi:10.3389/fpsyg.2015.00934)
Supplement: Supplementary file 1 [file SupplementaryMaterial.PDF]

Action dynamics reveal the impact of additional task factors on the execution of the prioritized motor movement in multitasking

Supplementary material

Stefan Scherbaum, Caroline Gottschalk, Maja Dshemuchadse, Rico Fischer

Department of Psychology, Technische Universität Dresden

Additional Results

Task 2: RT

We finally checked RT data of Task 2 for the expected effects (see Figure 3). A repeated measures analysis of variance (ANOVA) on RT with the factors $congruency_N$, $congruency_{N-1}$, and *SOA* revealed significant main effects for $congruency_N$, $F(1,19) = 80.07$, $p < 0.01$, $\eta^2_p = 0.81$ and *SOA*, $F(2,38) = 1125.638$, $p < 0.001$, $\eta^2_p = 0.98$, but no effect for $congruency_{N-1}$. Furthermore, there was a significant interaction $congruency_N$ x $congruency_{N-1}$, $F(1,19) = 99.36$, $p < 0.001$, $\eta^2_p = 0.84$. The main effect of *SOA* showed a linear trend with RT decreasing together with *SOA* $F(1,19) = 1413.73$, $p < 0.001$, $\eta^2_p = 0.99$.

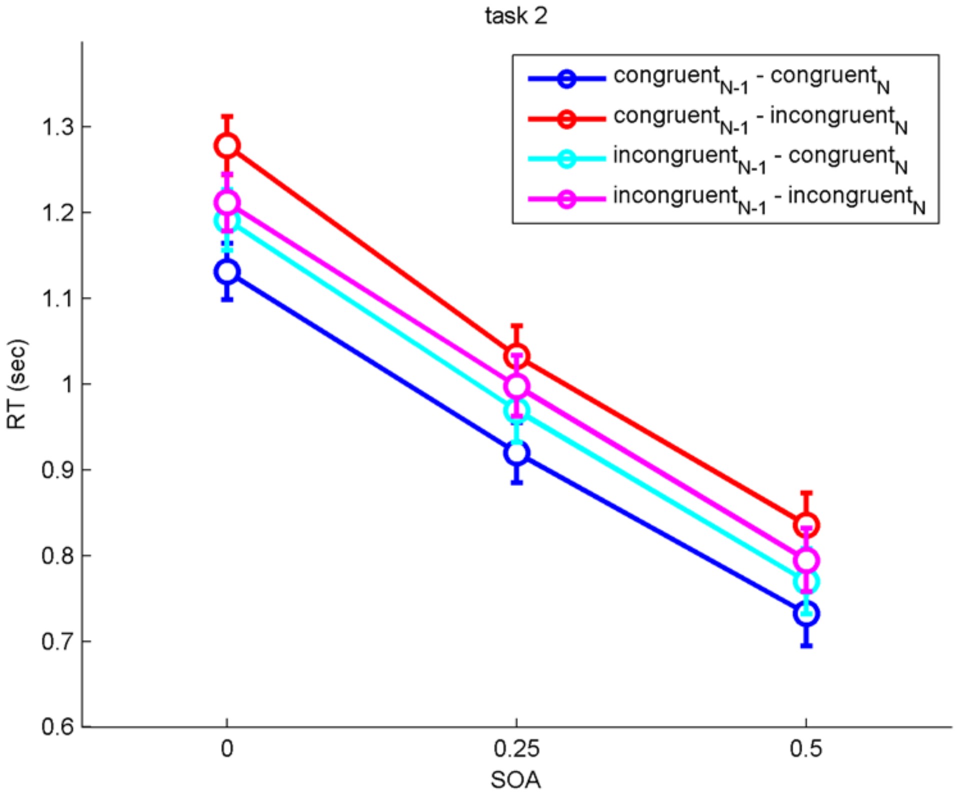

Figure 1. RT of task 2 as a function of SOA. Errorbars indicate standard-errors

Deflection of mouse movements

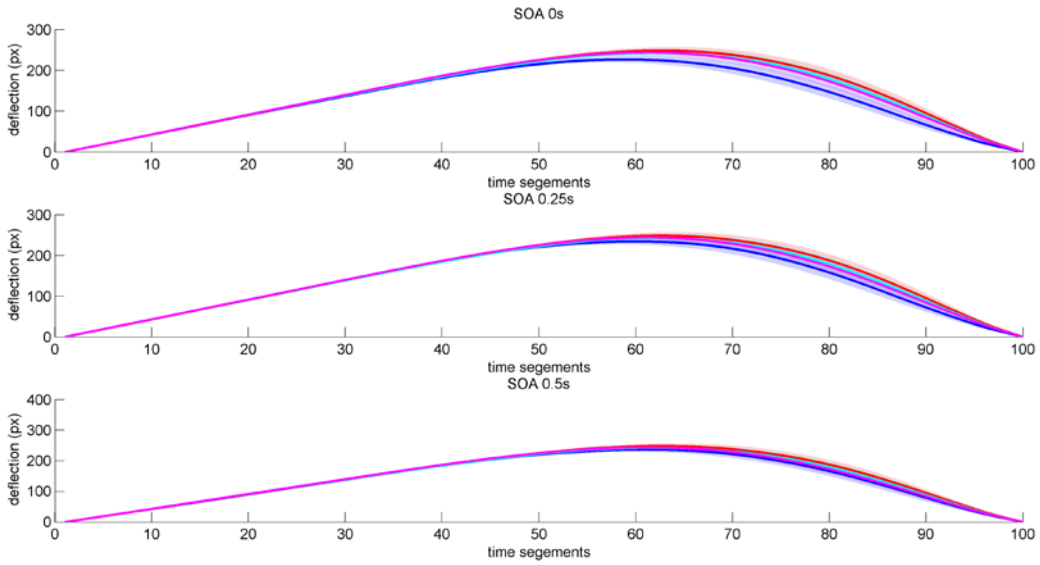

Figure 2. Deflection of mouse movements in Task 1 on the x-axis from the ideal direct path to the target box. Shaded areas indicate standard-errors.
