# Supplementary material for: Action dynamics in multitasking: the impact of additional task factors on the execution of the prioritized motor movement
Source: Front Psychol. 2015 Jul 6;6:934. doi: 10.3389/fpsyg.2015.00934 (PMC4491597; doi:10.3389/fpsyg.2015.00934)
Supplement: Supplementary file 2 [file Appendix.DOCX]

# Appendix

To establish a correction criterion for the use of multiple consecutive *t*-test on the results of continuous multiple regression analysis, we conducted monte carlo simulations on surrogate data based on the mean and standard deviations of the real data (strictly following the original approach of Dale et al., 2007).

We simulated 10000 synthetic experiments. In each of these experiments, we constructed synthetic congruent and incongruent trials for the condition SOA=500 ms (the smallest effect that could be expected in the data) for each participant. To create these synthetic congruent and incongruent trials, we sampled each time step from a normal distribution with the mean and standard deviation of this time step in the original congruent and incongruent trials. This procedure served to preserve the temporal dependence of the time step data in the synthetic data. Hence, for each synthetic experiment, we constructed for each participant datasets based on the real mean and standard deviations of the original data at each point in time.

We then applied the regression analysis for *congruency_N_* as on the real data and detected the longest segment of statistically significant *β*-weigths according to *t*-tests against zero. Based on 10000 simulated experiments, this yielded 10000 longest segments for which we calculated the distribution (see Table A 1).

Table A 1. Frequency (in %) of segment lengths from 10000 synthetic experiments

| Segment length | % | *p*-value |
| --- | --- | --- |
| 1 | 9 | 0,915 |
| 2 | 21 | 0,705 |
| 3 | 22 | 0,487 |
| 4 | 17 | 0,314 |
| 5 | 11 | 0,202 |
| 6 | 8 | 0,123 |
| 7 | 5 | 0,074 |
| **8** | **3** | **0,044** |
| 9 | 2 | 0,024 |
| 10 | 1 | 0,012 |
| **11** | **1** | **0,005** |

The simulation shows that segments of 8 or more consecutive significant time steps occur in less than 5% (*p* < 0.05) of cases and 11 or more consecutive time steps occur in less than 1% (*p* < 0.01) of cases. This establishes a criterion of 8-11 consecutive time steps for the analysis of the real data, of which we chose 10.

Descriptive data of one student participant was lost after data collection – hence, gender and age describe data of 19 participants. The experimental data are reported completely for all 20 participants.

At 92 Hz sampling frequency, 100 samples correspond to an RT of 1087 ms. This means that all trials below this RT, including trials of average RT (*M* = 682 ms, *SE* = 19 ms, 62 samples at 92Hz), are stretched to 100 samples. Only trials longer than 1087 ms (5.7% of all trials) are compressed.

For RT2, please see the supplementary material and the supplementary Figure 1

These analyses did not change qualitatively when removing all trials with RT lower than 500 ms.
